# Effects of time‐restricted feeding and meal timing on an 8‐week fat oxidation exercise training program—A randomized controlled trial

**DOI:** 10.14814/phy2.70194

**Published:** 2025-01-21

**Authors:** Florian Hofstätter, Martin Niedermeier, Linda K. Rausch, Martin Kopp, Lydia Simpson, Justin S. Lawley

**Affiliations:** ^1^ Department of Sport Science University of Innsbruck Innsbruck Austria; ^2^ Department of Physiology, Pharmacology and Neuroscience University of Bristol Bristol UK; ^3^ Institute of Mountain Emergency Medicine EURAC Research Bolzano Italy

**Keywords:** fat oxidation, fatmax training, meal timing, time restricted feeding

## Abstract

Time‐restricted feeding (TRF) and aerobic exercise are lifestyle interventions to prevent or manage different metabolic diseases. How these interventions interact, including the impact of meal timing, is not well understood. The aim of this study was to examine the influence of TRF on fat oxidation during exercise, whereby participants performed an 8‐week fat_max_‐training program either in the fasted state or after a carbohydrate‐based snack. 36 participants were randomized into three groups. (1) Training sessions were performed in the fasted state; (2) Training sessions were performed after consuming a standardized carbohydrate‐based snack; (3) Exercise training with an ad libitum diet as a control group. Pre‐ and post‐tests included anthropometric measurements and a fat_max_‐cycle‐ergometry protocol to measure substrate oxidation. Data were analyzed as workload‐matched and maximal fat oxidation using a series of mixed ANOVAs. Workload‐matched (*p* = 0.038) and maximal (*p* < 0.001) fat oxidation improved in all groups. No significant group × time interactions were found in substrate utilization. Time had a significant effect on body weight (*p* = 0.011), fat mass (*p* < 0.001), and muscle mass (*p* < 0.001). Results suggest that fat_max_ exercise training leads to improvements in fat oxidative capacity independent of fed or fasted state.

## 1 INTRODUCTION

Daily time‐restricted feeding (TRF) has been proposed as a therapeutic/beneficial lifestyle intervention in the management of obesity and metabolic disease (de Cabo & Mattson, 2019; Sun et al., 2024). Moreover, TRF is utilized in athletic populations to improve endurance performance (Moro et al., 2020). TRF, where daily caloric intake is restricted to a consistent time window, may exert its beneficial effects on metabolic health and/or exercise performance by reducing body weight through a reduction in calorie intake and fat mass (Anton et al., 2018; Moro et al., 2020). However, TRF may also improve metabolic health and/or performance independent of calorie restriction, including an increase in fat oxidation at rest and during aerobic exercise (Achten & Jeukendrup, 2004; Iwayama et al., 2015; Vieira et al., 2016). (Pan et al., 1997). In addition to TRF, aerobic exercise training is another lifestyle intervention shown to improve fat oxidation (Van Proeyen et al., 2011), and exercise training is widely implemented in athletes to improve fat oxidation (Achten & Jeukendrup, 2004; San‐Millán & Brooks, 2018). For example, Venables and Jeukendrup (2008) reported that a 4‐week aerobic training program, 30–60 min 5 d/week at an individualized intensity targeting maximal fat oxidation (i.e., fat_max_‐training), improves the rate of fat oxidation during exercise by 44% and was more effective in improving rate of fat oxidation than high‐intensity training (Venables & Jeukendrup, 2008). Thus, it is possible that combining TRF and fat_max_ training might be synergistic in increasing fat utilization during exercise, although, this has not been determined.

Importantly, while fat oxidation is high during light to moderate exercise (Achten et al., 2002), macronutrient intake prior to exercise modulates fuel utilization. Indeed, consuming a carbohydrate‐based meal leads to a rise in intramuscular malonyl–coenzyme A and suppression of CD36 and carnitine palmitoyl transferase‐1, which are key mitochondrial pathways responsible for beta‐oxidation of long‐chain fatty acids (Rasmussen & Wolfe, 1999). Thus, carbohydrate consumption prior to exercise prioritizes carbohydrate metabolism even during light‐ to moderate‐intense exercise (Achten & Jeukendrup, 2004; Erdmann et al., 2010). Prior studies have shown a suppression of fat oxidation after several weeks of carbohydrate‐fed aerobic training (Van Proeyen et al., 2011). Yet, while it is known that adipose tissue lipolysis increases in direct proportion to the duration of fasting (Montain et al., 1991), studies typically only include an overnight (~8 h) fast (Van Proeyen et al., 2011). Moreover, the carbohydrate feeding protocol is typically very high (160 g) in line with athletic performance and not reflective of a typical meal or snack that would be eaten by individuals looking to improve or maintain their health with fasting and exercise. Thus, while TRF and fat_max_ training may improve fat oxidation rates, these improvements may be blunted or ameliorated by simply eating a “typical” carbohydrate‐based snack prior to training sessions.

Therefore, the aim of this study was to examine the influence of a 16‐h TRF protocol and meal timing on fat oxidation during exercise, whereby healthy, recreationally active participants performed an 8‐week fat_max_‐training program and completed training either in the fasted state or after a carbohydrate‐based snack. Fat_max_ training with ad libitum food intake was used as a control group. We hypothesized that 8 weeks of fat_max_ training will increase the rate of fat oxidation during exercise, but this improvement in fat oxidation will be augmented when exercise training sessions are performed in the fasted state and attenuated in the fed state.

## 2 METHODS

### 2.1 Ethical approval

All participants provided written informed consent before enrollment in the study. The Physical Activity Readiness—Questionnaire was used to assess the physical activity readiness of study participants (Shephard et al., 1991).

### 2.2 Participants

Fifty‐two young, healthy, recreationally active students (76.9% female) were recruited from the University of Innsbruck via the university newsletter and personal contacts, with a total 36 completing the study (28 females, participant demographics presented in Table 1). All participants were free from chronic or acute disease and metabolic disorders and were not taking any prescription or over‐the‐counter medication at the time of participation. Participants were excluded if they had performed an intermittent fasting regime in the 3 months prior to the study.

**TABLE 1:** Participant demographics for each group after applying exclusion criteria.

	CG (*n* = 10)	FFG (*n* = 12)	FG (*n* = 14)	*p*‐Value	Effect sizes		
					CG – FFG	CG – FG	FFG – FG
Sex (m/f)	4/6	3/9	1/13	–	–	–	–
Age (years)	24.8 ± 5.1	24.2 ± 3.9	24.2 ± 3.4	0.922	0.156	0.145	0.012
Height (cm)	167.9 ± 5.7	171.2 ± 8.1	166.9 ± 4.7	0.217	0.529	0.154	0.683
Weight (kg)	68.2 ± 8.9	67.7 ± 13.9	62.1 ± 5.3	0.239*	0.050	0.618	0.568
BMI (kg/m^2^)	24.2 ± 3.3	22.9 ± 2.7	22.3 ± 1.8	0.203	0.522	0.751	0.229

*Note*: Data presented as mean ± standard deviations; *p*‐values represent ANOVA results; asterisks represent between‐group comparisons.

Abbreviations: CG, control group; FFG, fasted fed group; FG, fasted group.

*p ≤ 0.05, significant group difference between CG and FG; effect sizes are presented as Cohen's *d*.

### 2.3 Study design

The randomized controlled study consisted of three phases: a pre‐test phase, an intervention phase, and a post‐test phase. The pre‐ and post‐tests included anthropometric measurements (body weight, height, hip‐ and waist circumference), bio‐impedance analyses to assess relative fat and muscle mass, and an ergo‐spirometry exercise test to calculate each individual's maximal rate of fat oxidation (fat_max_ test). Prior to all tests, participants refrained from vigorous exercise for 48 h, fasted overnight, and were instructed to avoid caffeine intake for 12 h prior to the test. The menstrual phase was not controlled in female participants; however, all participants reported regular menstrual cycles between 28 and 35 days.

### 2.4 Anthropometric parameters

Body weight (BW) and height were measured using a body weight scale (Intelligent Body Fat Scale—Basic, Renpho EU, Spain) and a stadiometer (Seca Gmbh & Co. Kg., Germany). Furthermore, hip and waist circumferences were measured using a non‐elastic measuring tape (Smart Measuring Tape, Renpho EU, Spain), and body composition was determined via bio‐impedance analysis (BIA 101 Anniversary AKERN/RJL Systems, Italy), whereby the participants were advised to lie still for 15 min prior to the measurement to ensure stable, accurate values. All measurements were carried out in an overnight fasted state and after a bathroom break. A detailed diet diary was taken completed over the 24 h preceding the pre‐test visit, and participants were advised to repeat this diet on the day prior to the post‐test visit.

### 2.5 Assessment of maximal fat oxidation

The maximal rate of fat oxidation was assessed using a modified version of the fat_max_‐test protocol by Achten et al. (2002) on a cycle ergometer (Cyclus 2 Ergometer, RBM elektronik‐automation GmbH, Germany). After a 5‐min warm‐up (female 50 watts and male participants 80 watts), the workload was increased by 20 watts every 5 min until fat oxidation started to decline and respiratory exchange ratio (RER) of ≥1.0 was achieved for more than 30 s. Breath‐by‐breath oxygen consumption (V̇O_2_), carbon dioxide production (V̇CO_2_), and ventilation were measured via a metabolic gas analyzer (Metalyzer 3B, Cortex Biophysik GmbH, Germany), calibrated before each test. Heart rate data were measured using a chest strap (Polar, Finland) to identify training heart rate zones. Data for indirect calorimetry and heart rate were recorded continuously, and data were extracted over the final 2 min of each stage where VO2, VCO2, and ventilation were in a steady state.

### 2.6 Data processing

The primary outcome of this study was the maximal rate of fat oxidation (g∙min^−1^). The average V̇O_2_ and V̇CO2 were extracted over the final 2 min of each stage of the fat_max_‐test protocol, to ensure steady‐state values, and fat oxidation was calculated using the formula outlined by Péronnet & Massicotte (1991). The maximal rate of fat oxidation was taken as the workload prior to when fat oxidation started to decline with increasing intensity. The rate of carbohydrate oxidation and total energy expenditure were determined at the same time points utilizing the formulae established by Péronnet & Massicotte (1991).

$$\text{Total}\;\mathrm{fat}\;\text{oxidation}\;\left(g\;\min^{-1}\right)=1.695\times \dot{V}O_{2}–1.701\times \dot{V}\text{CO2}.$$

$$\text{Total}\;\mathrm{CHO}\;\text{oxidation}\;\left(g\;\min^{-1}\right)=4.210\times \dot{V}{\mathrm{CO}}_{2}–2.9621\times \dot{V}O_{2}.$$

Importantly, the exercise training and/or the TRF intervention might improve the maximal rate of fat oxidation (i.e., the absolute ability to use fat as a fuel during exercise) or simply shift the maximal fat oxidation rate to a higher workload (i.e., no change in the maximal rate of fat oxidation, but the same rate of fat oxidation would occur at a higher workload) indicating a more efficient use of fat as a fuel. Thus, data were extracted and fat oxidation is presented as workload‐matched (i.e., the same absolute workload pre‐ and post‐intervention) and maximal (i.e., the highest rate of fat oxidation independent of workload) intensities.

### 2.7 Exercise training

The primary outcome of this training intervention was to improve fat oxidation during exercise. Thus, aerobic exercise training was performed at an individualized intensity associated with maximal fat oxidation, which has been shown to improve the rate of fat oxidation to a greater extent than high‐intensity training (Venables & Jeukendrup, 2008). Therefore, all three groups performed a supervised 8‐week training intervention consisting of cycling 3×/week for 60 min at their individual fat_max_ zone heart rate (90%–100% of fat_max_ heart rate identified in the pre‐test exercise test) (Achten et al., 2002). Heart rate was measured (PM100, Medisana, part of Ogawa Smart Healthcare Technology Group Co. Ltd., China) during each training session and the external workload was adjusted over the 8 weeks to maintain each participant's pre‐identified heart rate zone, thus ensuring consistent internal workload and progressive overload training. Training hours for all groups were between 08:00 am and 01:00 pm on weekdays.

### 2.8 Fasting regime

Participants were randomized into one of three groups: (1) a TRF window of 8 h (i.e., 16‐hour fast) with all training sessions performed at the end of the fasting window (fasting group, FG), no provided snack before or after the training session; (2) a TRF window of 8 hours (i.e., 16‐h fast), with all training sessions performed after consuming a small standardized carbohydrate‐based snack (two bars equal 50 g, 212 kcal, fat 6.5 g, carb 33.5 g, fiber 1.9 g, protein 3.9 g, salt 0.25 g) (fasting but trained fed group, FFG); and (3) participants undertook exercise training but were asked to follow their typical ad libitum diet (control group, CG). The CG was not given instructions what to eat prior to their exercise training sessions. For the fasting groups FG and FFG, fasting hours were documented with a diet diary completed daily. The timing of the fasting window was not prescribed to avoid any potential effects on circadian rhythm (Wehrens et al., 2017) and instead were synced to the participants desired training schedule with a constant schedule maintained over the 8 weeks.

### 2.9 Power calculation

At the time of the study, we were not aware of any studies reporting effects of combining time‐restricted feeding and fat_max_ exercise training during a multi‐week intervention on substrate utilization. Thus, no a priori sample size was calculated for the present study. However, participants were recruited for a larger‐scale project including acute and chronic psychological parameters; therefore, power calculation was conducted for the main psychological parameter Positive and Negative Affective Scale (Watson & Clark, 1988), resulting in a final sample size of *n* = 52 (including an estimated dropout rate of 20%).

### 2.10 Statistical analysis

Participant compliance to the fasting intervention was compared between FFG and FG groups with Student's independent sample *t*‐test. Moreover, the average heart rates over the 8‐week training intervention were compared between groups using a single between‐subject factor (group: CG, FG, FFG) ANOVA. Upon a significant main effect, follow‐up comparisons were conducted with Bonferroni corrections.

Outcome variables were statistically analyzed using a series of 3 × 2 mixed ANOVAs. The between‐subject factor group contained three levels (CG, FG, FFG), and the within‐subject factor time contained two levels (pre‐test, post‐test). Upon significant main effect or interactions, follow‐up comparisons were conducted with Bonferroni corrections. Moreover, as the primary question for the current study concerns changes in substrate utilization between groups, the change score for fat, CHO, EE, HR between pre‐ and post‐test was calculated for each variable and statistically analyzed using a series of one‐way ANOVAs with a between‐subject factor of group contained 3 levels (CG, FG, FFG). Upon significant main effect or interactions, follow‐up comparisons were conducted with Bonferroni corrections. Effect sizes are presented as partial eta squared for 3 × 2 mixed ANOVAs, and Cohen's *d* for group comparisons of one‐way ANOVAs. All data were analyzed with Jamovi v2.3 (The Jamovi Project, 2022) with the threshold for statistical significance set at ≤0.05 (two‐tailed).

## 3 RESULTS

### 3.1 Exercise training

Individuals with a compliance rate of <80% for exercise training and/or adherence of <80% for the fasting regime were excluded from data analysis. Out of 52 participants, 15 did not complete the intervention phase, and one participant had an exercise compliance <60% (total dropout rate 30.7%, CG *n* = 4, FFG *n* = 5, FG *n* = 6). Therefore, 36 participants (28 females, 8 males; age 24.4 ± 3.9 years; height 168.6 ± 6.4 cm; weight 65.7 ± 10.0 kg; body mass index 23.0 ± 2.6 kg/m^2^) were included in the final analysis (Table 1). Compliance rates of both the FFG and FG groups were similar (*p* = 0.279) and all groups attended a similar number of training session (*p* = 0.417), with training performed at a similar internal training load (*p* = 0.564), see Table 2. Absolute values for metabolic and heart rate data for all groups are presented in Table 3.

**TABLE 2:** Study compliance, number of training sessions, and heart rate for each group after apply exclusion criteria.

	CG (*n* = 10)	FFG (*n* = 12)	FG (*n* = 14)	*p*‐Value	Effect sizes		
					CG – FFG	CG – FG	FFG – FG
Fasting compliance (%)	n.a.	89.4 ± 6.2	92.1 ± 6.0	0.279	n.a.	n.a.	0.436
Exercise compliance (%)	88.3 ± 7.6	87.5 ± 5.3	85.1 ± 5.6	0.397	0.139	0.534	0.395
Total training sessions (*n*)	21.2 ± 1.8	21.0 ± 1.2	20.4 ± 1.3	0.397	0.138	0.533	0.395
Fat_max_‐HR	142.8 ± 10.8	140.3 ± 15.2	146.2 ± 12.6	0.519	0.193	0.259	0.452

*Note*: Data presented as mean ± standard deviations; *p*‐values represent ANOVA results. Effect sizes are presented as Cohen's *d*.

Abbreviations: CG, control group; FFG, fasted fed group; FG, fasted group; n.a., not applicable.

**TABLE 3:** Absolute data for metabolic and heart rate for all groups.

	CG			FFG			FG		
	Pre	Matched	Absolute	Pre	Matched	Absolute	Pre	Matched	Absolute
Fat (g∙min^−1^)	0.44 ± 0.14	0.52 ± 0.13	0.56 ± 0.13	0.35 ± 0.17	0.36 ± 0.08	0.39 ± 0.08	0.34 ± 0.15	0.39 ± 0.10	0.43 ± 0.11
CHO (g∙min^−1^)	1.12 ± 0.37	0.80 ± 0.45	1.08 ± 0.41	1.03 ± 0.53	0.81 ± 0.42	0.91 ± 0.33	0.98 ± 0.36	0.66 ± 0.23	0.90 ± 0.24
EE (kcal∙min^−1^)	8.91 ± 2.01	8.35 ± 1.73	9.95 ± 2.05	7.70 ± 2.73	6.84 ± 2.26	7.59 ± 1.48	7.30 ± 0.90	6.57 ± 0.84	7.92 ± 1.06
HR (beats∙min^−1^)	143 ± 10.8	131 ± 8.3	144 ± 10.7	140 ± 15.2	127 ± 15.4	136 ± 15.1	146 ± 12.9	130 ± 11.7	145 ± 13.0

*Note*: Data presented as mean ± standard deviations. Effect sizes are presented as Cohen's *d*.

Abbreviations: CG, control group; CHO, carbohydrate oxidation rate; EE, energy expenditure; Fat, Fat oxidation rate; FFG, fasted fed group; FG, fasted group; HR, Heart rate.

3 × 2 mixed ANOVA showed a significant main effect of time in body weight, BMI, relative fat mass (rel. FM), and relative muscle mass (rel. MM) (BW: *p* = 0.011, $\eta_{p}^{2}$ = 0.180; BMI: *p* = 0.011, $\eta_{p}^{2}$ = 0.179; rel. FM: *p* < 0.001, $\eta_{p}^{2}$ = 0.522; rel. MM: *p* < 0.001, $\eta_{p}^{2}$ = 0.295), when all groups were combined. Significant group‐by‐time differences were only apparent in body weight, BMI, and waist circumference (body weight: *p* = 0.002, $\eta_{p}^{2}$ = 0.319; BMI: *p* = 0.001, $\eta_{p}^{2}$ = 0.326; waist: *p* = 0.034, $\eta_{p}^{2}$ = 0.186). CG and FFG showed decreases, whereas FG showed increases (Table 4).

**TABLE 4:** Change scores (Δ) of anthropometrics calculated from pre‐ to post‐test.

	Δ CG	Δ FFG	Δ FG	*p*‐Value	Effect size*s*		
					CG – FFG	CG – FG	FFG – FG
Body weight (kg)	−0.64 ± 0.75	−1.90 ± 2.29	0.46 ± 1.03	0.002*	0.827	0.719	1.546
BMI (kg/m^2^)	−0.22 ± 0.26	−0.63 ± 0.74	0.16 ± 0.36	0.001*	0.820	0.751	1.571
Hip (cm)	0.05 ± 3.59	−1.52 ± 3.34	−0.56 ± 3.10	0.538	0.472	0.183	0.289
Waist (cm)	−0.96 ± 3.12	−1.52 ± 2.79	1.01 ± 1.46	0.034*	0.226	0.798	1.024
FM (%BW)	−1.80 ± 3.08	−3.58 ± −7.00	−1.86 ± −6.00	0.133	0.747	0.024	0.723
MM (%BW)	1.00 ± 2.31	2.17 ± 1.90	0.57 ± 1.83	0.132	0.585	0.215	0.800

*Note*: Data presented as mean ± standard deviations; asterisks represent between‐group comparisons.

Abbreviations: BMI, body mass index; BW, body weight; CG, control group; FFG, fasted fed group; FG, fasted group; FM, fat mass; MM, muscle mass.

*p ≤ 0.05, significant difference between FFG versus FG; effect sizes are presented as Cohen's *d*.

### 3.2 Exercise variables at workload‐matched intensity

**FIGURE 1:**
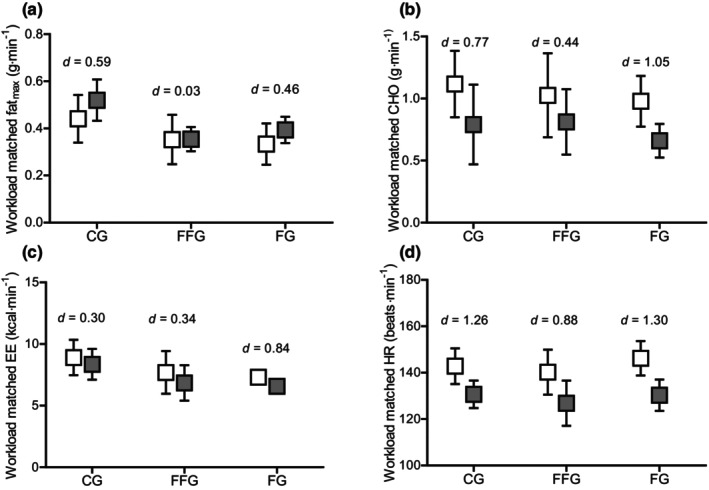
Comparison of workload‐matched values from pre‐ to post‐test split by groups. (a) Workload matched fat oxidation; (b) workload matched CHO oxidation; (c) workload matched EE; (d) workload matched HR. Boxes represent means; error bars represent 95% confidence intervals; white and gray boxes represent pre‐test and post‐test, respectively; CHO, carbohydrates; EE, energy expenditure; HR, heart rate; within‐group effect sizes are presented as Cohen's d.

Pre‐ to post‐intervention, fat oxidation at the workload‐matched intensity was elevated in all groups (*p* = 0.038, $\eta_{p}^{2}$ = 0.124), whereas CHO‐oxidation decreased significantly in all groups (*p* < 0.001, $\eta_{p}^{2}$ = 0.496) according to the main effect of time in the ANOVA. Energy expenditure (EE) and heart rate (HR) decreased at the workload‐matched intensity (EE: *p* < 0.001, $\eta_{p}^{2}$ = 0.552; HR: *p* < 0.001, $\eta_{p}^{2}$ = 0.717) (pre‐ to post‐changes scores, see Table 5). However, no statistically significant group‐by‐time interaction effects were observed for any workload‐matched parameters (fat_max_: *p* = 0.371, $\eta_{p}^{2}$ = 0.058; CHO: *p* = 0.540, $\eta_{p}^{2}$ = 0.037; EE: *p* = 0.470, $\eta_{p}^{2}$ = 0.045; HR: *p* = 0.570, $\eta_{p}^{2}$ = 0.033), indicating the effects were not different between groups, see Figure 1.

**TABLE 5:** Change scores (Δ) for metabolic and heart rate data calculated from pre‐ to post‐test at the workload‐matched intensity.

	Δ CG	Δ FFG	Δ FG	*p*‐Value	Effect size*s*		
					CG – FFG	CG – FG	FFG – FG
Fat (g∙min^−1^)	0.08 ± 0.13	0.00 ± 0.13	0.06 ± 0.13	0.377	0.578	0.154	0.424
CHO (g∙min^−1^)	−0.32 ± 0.28	−0.21 ± 0.31	−0.31 ± 0.30	0.630	0.365	0.020	0.344
EE (kcal∙min^−1^)	−0.56 ± 0.75	−0.86 ± 0.60	−0.73 ± 0.51	0.614	0.485	0.275	0.210
HR (beats∙min^−1^)	−12.10 ± 9.70	−13.42 ± 7.09	−15.93 ± 9.85	0.625	0.147	0.426	0.280

*Note*: Data presented as mean ± standard deviations; *p*‐value shows ANOVA results. Effect sizes are presented as Cohen's *d*.

Abbreviations: CG, control group; CHO, carbohydrate oxidation rate; EE, energy expenditure; Fat, fat oxidation rate; FFG, fasted fed group; FG, fasted group; HR, heart rate.

### 3.3 Exercise variables at the maximal intensity

**FIGURE 2:**
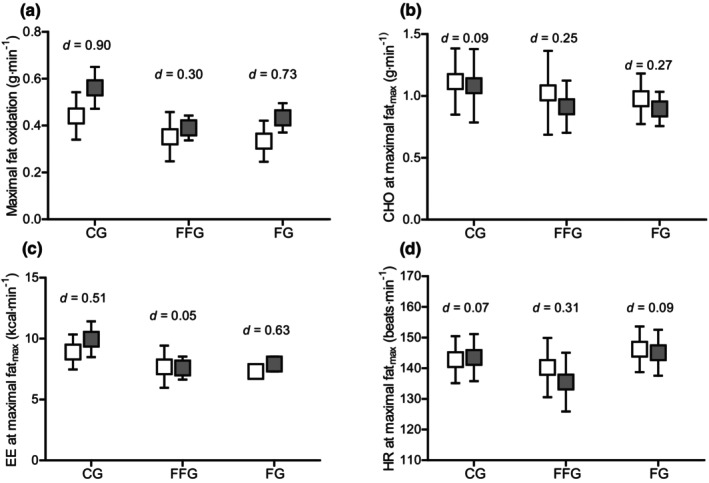
Comparison of maximal values at fat_max_‐workload from pre‐ to post‐test split by groups. (a) Maximal fat oxidation; (b) CHO oxidation at fat_max_; (c) EE at fat_max_; (d) HR at fat_max_. Boxes represent means, error bars represent 95% confidence intervals; white and gray boxes represent pre‐test and post‐test, respectively; CHO, carbohydrates; EE, energy expenditure; HR, heart rate; within‐group effect sizes are presented as Cohen's *d*.

Pre‐ to post‐intervention, the maximal fat oxidation rate increased (*p* < 0.001, $\eta_{p}^{2}$ = 0.310), when all groups were combined, although CHO oxidation was not different (*p* = 0.269, $\eta_{p}^{2}$ = 0.037). Energy expenditure at the post‐test fat_max_ increased significantly (*p* = 0.021, $\eta_{p}^{2}$ = 0.152) due to the greater absolute workload achieved post‐test in all groups. Heart rate at fat_max_ did not change significantly (*p* = 0.445, $\eta_{p}^{2}$ = 0.018) (pre‐ to post‐change scores, see Table 6). No significant group‐by‐time interactions at the maximal fat oxidation rate were observed (fat_max_: *p* = 0.262, $\eta_{p}^{2}$ = 0.078; CHO: *p* = 0.966, $\eta_{p}^{2}$ = 0.002; EE: *p* = 0.111, $\eta_{p}^{2}$ = 0.125; HR: *p* = 0.618, $\eta_{p}^{2}$ = 0.029), see Figure 2.

**TABLE 6:** Change scores (Δ) for metabolic and heart rate data calculated from pre‐ to post‐test at the maximal fat_max_ intensity.

	Δ CG	Δ FFG	Δ FG	*p*‐Value	Effect sizes		
					CG – FFG	CG – FG	FFG – FG
Fat (g∙min^−1^)	0.12 ± 0.12	0.04 ± 0.14	0.10 ± 0.14	0.350	0.614	0.184	0.430
CHO (g∙min^−1^)	−0.03 ± 0.26	−0.11 ± 0.52	−0.08 ± 0.47	0.888	0.176	0.107	0.069
EE (kcal∙min^−1^)	1.04 ± 0.94	−0.11 ± 1.69	0.62 ± 1.12	0.161	0.884	0.325	0.559
HR (beats∙min^−1^)	0.70 ± 10.39	−4.75 ± 17.62	−1.14 ± 10.60	0.679	0.409	0.138	0.271

*Note*: Data presented as mean ± standard deviations; *p*‐value shows ANOVA results; asterisks represent between‐group comparisons. Effect sizes are presented as Cohen's *d*.

Abbreviations: CG, control group; CHO, carbohydrate oxidation rate; EE, energy expenditure; Fat, fat oxidation rate; FFG, fasted fed group; FG, fasted group; HR, heart rate.

### 3.4 Exercise variables at the workload‐matched intensity

**FIGURE 3:**
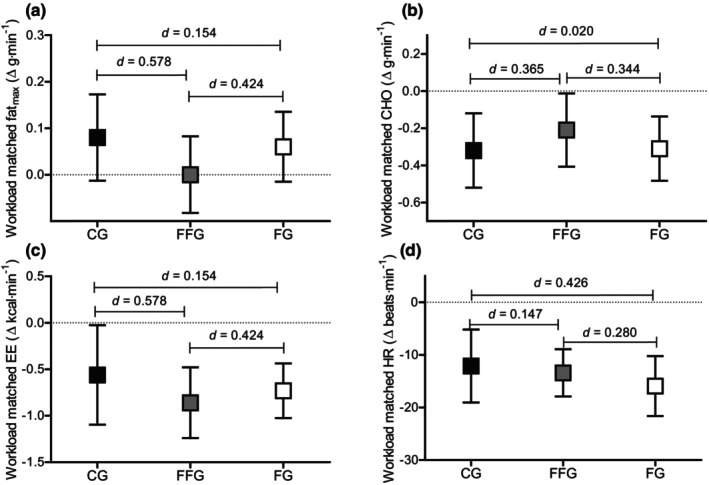
Comparison of change scores from pre‐ to post‐test at workload‐matched intensity split by groups. (a) Workload matched fat oxidation; (b) workload matched CHO oxidation; (c) workload matched EE; (d) workload matched HR. Boxes represent means; error bars represent 95% confidence intervals; black, gray and white boxes represent CG, FFG, and FG, respectively; CHO, carbohydrates; EE, energy expenditure; HR, heart rate; between‐group effect sizes are presented as Cohen's *d*.

No significant differences were observed in any exercise variables when pre‐to‐post data were compared as a change score for the workload‐matched trials. However, effect sizes did suggest a non‐significant increase in workload‐matched fat oxidation in the CG (*d* = 0.59) and FG (*d* = 0.46) compared to the FFG (*d* = 0.03), see Figure 3.

### 3.5 Exercise variables at the maximal intensity

**FIGURE 4:**
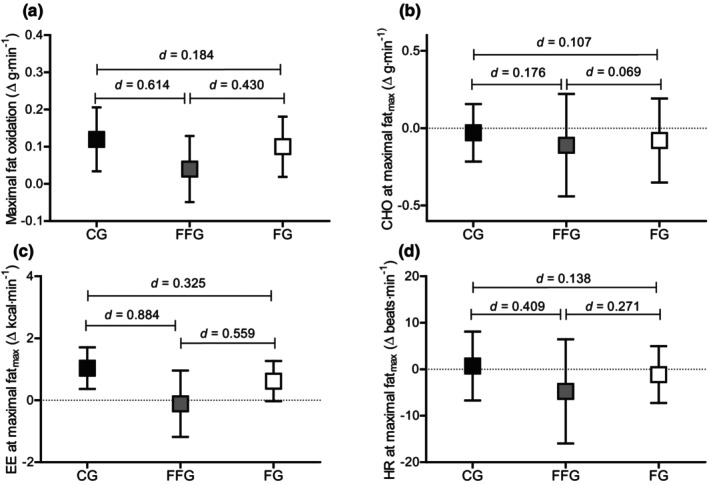
Comparison of change scores from pre‐ to post‐test at maximal fat_max_ intensity split by groups. (a) Maximal fat oxidation; (b) CHO oxidation at fat_max_; (c) EE at fat_max_; (d) HR at fat_max_. Boxes represent means, error bars represent 95% confidence intervals; black, gray and white boxes represent CG, FFG, and FG, respectively; CHO, carbohydrates; EE, energy expenditure; HR, heart rate; between‐group effect sizes are presented as Cohen's *d*.

Analyses of variance showed no significant differences in mean pre‐to‐post change scores at the intensity where maximal rate of fat oxidation was observed between groups. However, effect sizes indicated that change scores for maximal fat oxidation were higher in the CG (*d* = 0.90) and FG (*d* = 0.73) post‐test compared to the FFG (*d* = 0.30), see Figure 4.

## 4 DISCUSSION

Manipulating diet and/or incorporating aerobic exercise training are modifiable lifestyle interventions to increase the contribution of fat to substrate utilization. The main outcome of the current study was that an 8‐week fat_max_‐training program increased the rate of fat oxidation during exercise in healthy young individuals. Yet, the addition of TRF and exercising in the fasted state had no additional benefit. While not reaching the threshold for statistical significance, mean values and effect sizes imply that eating a carbohydrate‐based snack prior to each training session blunted the improvement in exercise fat oxidation despite the addition of a 16‐h fasting window.

### 4.1 Fat_max_ training improves fat oxidation during exercise

Our data support previous studies that exercise training at an intensity that elicits maximum fat oxidation rates improves the rate of fat oxidation at a workload‐matched intensity and increases the maximum rate of fat oxidation (Achten & Jeukendrup, 2004; Venables & Jeukendrup, 2008). Obesity and/or metabolic syndrome are associated with an increase in intramyocellular lipids or intramyocellular triglycerides concentrations that are linked to insulin resistance (Goodpaster et al., 2000). Interestingly, greater fat oxidation rates after a period of low‐intensity training stems predominantly from non‐plasma fatty acid oxidation (i.e., likely the skeletal muscle (Van Aggel‐Leijssen et al., 2002)). Therefore, fat_max_ training, which is generally at a relatively low‐intensity in untrained individuals (Venables & Jeukendrup, 2008), seems to be an attractive lifestyle intervention, even for young and relatively healthy individuals to prevent the excessive accumulation of intramuscular triglyceride concentrations and potentially prevent insulin resistance. However, it is worth noting that paradoxically, aerobic exercise training actually increases the number of intramyocellular lipids, which are also relatively higher in trained athletes (Howald et al., 2002). Thus, an important distinction may be that exercise training reduces the droplet size of intramyocellular lipids alongside an increase in mitochondrial content (increased mitochondria size) such that there is an increase in the overall area of intramyocellular lipids in contact with the mitochondria, which may improve overall fat oxidation (Tarnopolsky et al., 2007).

### 4.2 Time restricted feeding and exercising in the fasted state does not augment improvements in fat oxidation after 8‐weeks of fat_max_ training

In the current study, the addition of a 16‐h fasting window did not provide any additional improvements in fat oxidation rates beyond an ad libitum control group. This is surprising as both low‐intensity training (Achten & Jeukendrup, 2004; Venables & Jeukendrup, 2008) and TRF (Jong‐Yeon et al., 2002) have independently been shown to improve fat oxidation, and this seems to be independent of body fat percentage (Blaize et al., 2014; Kerhervé et al., 2020). One potential explanation is that both exercise training and TRF might improve fat oxidation through a similar rate‐limiting mechanism. For example, previous experiments have identified that carnitine palmitoyltransferase‐1 and FAT/CD36 on the mitochondria are important rate‐limiting steps in fatty acid oxidation (Bezaire et al., 2006). Carnitine palmitoyltransferase‐1 activity is elevated in trained individuals (Starritt et al., 2000) and both carnitine palmitoyltransferase‐1 activity (Bruce et al., 2006) and FAT/CD36 colocalization with carnitine palmitoyltransferase‐1 can be improved with aerobic training (Schenk & Horowitz, 2006). Moreover, fasting increases the transcription of carnitine palmitoyltransferase‐1 in skeletal muscle (Pilegaard et al., 2003). Thus, it is possible that tailored fat_max_ training may maximize improvements in biochemical pathways linked with skeletal muscle fat oxidation over 8 weeks such that no further improvements can be obtained from the addition of TRF. A similar explanation might be that low‐intensity aerobic training is known to improve mitochondrial content and their interaction with intramyocellular lipids (see discussion above), which may dominate improvements in fat oxidation beyond enzyme‐mediated changes in fat oxidation from TRF. We are not aware of any evidence that TRF alone can increase mitochondrial content in human skeletal muscle.

### 4.3 Time restricted feeding and exercising after a carbohydrate‐based snack tentatively suppresses improvements in fat oxidation after 8 weeks of fat_max_ training

Several prior investigations have clearly shown that acute carbohydrate feeding prior to or during aerobic exercise influences fat metabolism measured at the cellular (Bergman et al., 1999; Civitarese et al., 2005; Cluberton et al., 2005) and whole‐body level—even at intensities that typically elicit maximal fat oxidation (Achten & Jeukendrup, 2003). Moreover, 6 weeks of aerobic (~70% V̇O_2_max) training in the fed state (~160 g of carbohydrate) chronically altered cellular pathways indicative of fat metabolism and reduced the improvement in the maximal rate of fat oxidation compared to an overnight (8 h) fasting group. Albeit, fed training did not affect fat oxidation during a workload‐matched intensity. Our data utilizing a more realistic carbohydrate ingestion (~34 g) for the general public prior to each training session also implies that fed exercise may suppress improvements in fat oxidation (workload‐matched and maximal oxidation rates) despite a long fasting window (16 h) and a training program tailored to improve fat oxidation. Indeed, both the CG and the FG improved the maximal rate of fat oxidation by ~27%, whereas the FFG improved by only 11% with moderate to large effect sizes (CG vs. FFG, d = 0.614; FFG vs. FG, d = 0.430, see Table 6). Hypothetically, the pre‐exercise carbohydrate snack could have reduced the oxygen cost of exercise and reduced the absolute training intensity in the FFG group. However, heart rate data suggests that all groups trained at a similar internal workload (Table 2) as acute feeding does not seem to cause major shift is the oxygen cost of exercise (Astorino et al., 2019; Rodrigues et al., 2019).

### 4.4 The combination of time restricted feeding and exercise training in the fasted state resulted in a suppressing of weight loss compared to exercise in the fed state

Significant group‐by‐time interactions were observed in body weight, BMI, and waist circumference. However, these data may seem controversial as only the CG and FFG lost body weight and reduced their waist circumference, while the FG gained weight and their waist circumference increased. It is not entirely clear why the FG gained weight as several studies have reported beneficial effects of TRF with or without additional exercise on body composition (Moro et al., 2016; Tinsley et al., 2017; Tinsley & La Bounty, 2015). While speculative, we propose that the fasting groups might have intentionally or unintentionally consumed an excessive number of calories during their feeding windows (i.e., compensatory eating). However, the shorter feeding window experienced by the FFG (with the 8‐h feeding window further reduced by the time between the pre‐exercise meal and the end of the exercise session) may have mitigated this effect Table 7.

**TABLE 7:** Absolute data for anthropometrics for all groups.

	CG		FFG		FG	
	Pre	Post	Pre	Post	Pre	Post
Weight (kg)	68.2 ± 8.86	67.6 ± 8.71	67.7 ± 13.90	65.8 ± 13.30	62.1 ± 5.29	62.6 ± 5.61
BMI (kg/m^2^)	24.2 ± 3.30	24 ± 3.32	22.9 ± 2.65	22.3 ± 2.59	22,3 ± 1.75	22,5 ± 1.82
Hip (cm)	99.4 ± 7.84	99.5 ± 7.06	99.6 ± 5.92	98.1 ± 6.44	99.8 ± 4.10	99.3 ± 5.56
Waist (cm)	77.8 ± 8.14	76.8 ± 8.49	75.6 ± 9.92	74.1 ± 9.37	70.7 ± 3.65	71.8 ± 4.22
FM (% BW)	23.4 ± 7.71	21.6 ± 7.50	25.8 ± 6.47	22.2 ± 7.72	25.9 ± 5.41	24.1 ± 5.70
MM (% BW)	55.6 ± 6.85	56.6 ± 6.67	50.6 ± 4.78	52.8 ± 6.06	51.2 ± 4.06	51.8 ± 4.41

*Note*: Data presented as mean ± standard deviations; asterisks represent between‐group comparisons. Effect sizes are presented as Cohen's *d*.

Abbreviations: BMI, body mass index; CG, control group; FFG, fasted fed group; FG, fasted group; FM, fat mass; MM, muscle mass.

## 5 LIMITATIONS

The major limitation of the current study was our inability to control the macronutrient intake in all three groups and our reliance on self‐reported diet diaries in the fasting groups. Albeit this is a typical limitation of most studies involving time‐restricted feeding or fasting. It is also noteworthy that this study focused on young, healthy participants with the goal of maximizing rate of fat oxidation. These results cannot easily be translated to patient populations aiming to reverse or attenuate disease progression and this should be the focus of future studies. Another limitation is lack of statistical power for the comparison of fat oxidation in the FFG group. Despite a prior power calculation, the analysis of variance displayed a significant main effect of time without a statistically significant interaction effect implying all three groups improved exercise fat oxidation to an equal extent. However, in our opinion, the absolute values, the individual (pre‐post) effect sizes and the effect sizes for the change scores highlight that the CG and FG groups had a similar improvement in exercise fat oxidation in contrast to almost no change in the FFG. We appreciate this comparison did not reach the classic statistical threshold and thus we advise the reader that caution is warranted with regard to our interpretation of the data. Finally, another limitation is that all our outcome variables were performed in the fasted state, which may not reflect normal activities of daily living. Indeed, after consuming a meal, especially containing carbohydrate, the identified benefits of fat_max_ training may be ameliorated. Future research is needed to examine this effect.

## 6 CONCLUSION

Conclusions of the current study are that fat_max_ training is an effective lifestyle intervention to improve fat oxidation in young healthy individuals. Moreover, while the addition of a 16‐h TRF protocol did not enhance exercise fat oxidation, it neither hindered the uptake of such a training protocol as we observed similar compliance rates (~85%) over the 8 weeks of training in all three groups. Conversely, eating a small carbohydrate snack prior to each exercise session seemed to suppress improvements in exercise fat oxidation despite the addition of a 16‐h fasting window. Collectively, these data suggest fat_max_ training might be an effective training intervention in healthy individuals looking to improve their maximum rate of fat oxidation, regardless of the fed state.

## AUTHOR CONTRIBUTIONS

**Florian Hofstätter:** conceptualization, methodology, formal analysis, investigation, writing—original draft, visualization, project administration, funding acquisition. **Linda K Rausch:** writing—review and editing. **Martin Niedermeier:** formal analysis, writing—review and editing. **Martin Kopp:** writing—review and editing, supervision. **Lydia Simpson:** formal analysis, writing—review and editing. **Justin S. Lawley:** writing—review and editing, supervision.

## FUNDING INFORMATION

This study was partly funded by a grant from the University of Innsbruck's Young Investigator Program.

## CONFLICT OF INTEREST STATEMENT

The authors declare that they have no known competing financial interests or personal relationships that could have appeared to influence the work reported in this paper.

## ETHICS STATEMENT

This study was reviewed by the University Ethics Board at Innsbruck University (#91/2021) and conformed to the ethical principles and guidelines of good scientific practice stated in the Declaration of Helsinki, except for registration in a database.
